# Supplementary material for: Novel Genetically Encoded Bright Positive Calcium Indicator NCaMP7 Based on the mNeonGreen Fluorescent Protein
Source: Int J Mol Sci. 2020 Feb 28;21(5):1644. doi: 10.3390/ijms21051644 (PMC7084697; doi:10.3390/ijms21051644)
Supplement: Supplementary file 1 [file ijms-21-01644-s001.zip › Supplementary Information/Supplementary information.pdf]

Supplementary Information

Novel genetically encoded bright positive calcium indicator NCaMP7 based on the mNeonGreen fluorescent protein

Oksana M. Subach, Vladimir P. Sotskov, Viktor V. Plusnin, Anna M. Gruzdeva, Natalia V. Barykina, Olga I. Ivashkina, Konstantin V. Anokhin, Alena Y. Nikolaeva, Dmitry A. Korzhenevskiy, Anna V. Vlaskina, Vladimir A. Lazarenko, Konstantin M. Boyko, Tatiana V. Rakitina, Anna M. Varizhuk, Galina E. Pozmogova, Oleg V. Podgorny, Kiryl D. Piatkevich, Edward S. Boyden, and Fedor V. Subach

Supplementary Figures and Tables

**Table S1**	**2**
**Table S2**	**3**
**Table S3**	**4**
**Table S4**	**5**
**Table S5**	**6**
**Table S6**	**7**
**Table S7**	**8**
**Table S8**	**9**
**Figure S1**	**10**
**Figure S2**	**11**
**Figure S3**	**12,13**
**Figure S4**	**14**
**Figure S5**	**15**
**Figure S6**	**16**
**Figure S7**	**17**
**Figure S8**	**18**
**Figure S9**	**19**
**Figure S10**	**20**
**Figure S11**	**21**
**Figure S12**	**22**
**Figure S13**	**23**
**Figure S14**	**24**
**Figure S15**	**25**
**Figure S16**	**26**
**Figure S17**	**27,28**
**Video S1**	**29**
**Video S2**	**30**
**Supplementary references**	**31**

Table S1. ***In vitro*** **properties for the purified derivatives of NCaMP7 indicator with different calcium affinities.**

Properties		Proteins					
		NCaMP4		NCaMP9		NCaMP10	
		apo	sat	apo	sat	apo	sat
Abs/Exc maximum (nm)		400/400	505/508	403/402	509/512	403/408	509/512
Emission maximum (nm)		520		520	522	520	522
Quantum yield [a]		0.079±0.002	0.53±0.03	0.047±0.003	0.51±0.03	0.056±0.002	0.52±0.03
ε (mM^{-1} cm^{-1}) [b]		43.3±5.9	101.0±4.0	51.2±4.8	106.5±6.2	46.0±6.2	112.0±5.5
Brightness (%) [c]		11	166	7.4	170	8.1	182
ΔF/F (fold)	0 mM Mg^{2+}	32±5		99±73		61±16	
	1 mM Mg^{2+}	8.9±0.2		29±5		15±1	
pK_a		5.38±0.03<br>6.84±0.04	5.88±0.16	5.43±0.09<br>6.58±0.15	6.04±0.30	5.44±0.09<br>6.61±0.1	6.15±0.29
Kd (nM)[d]	0 mM Mg^{2+}	66±4<br>[n=1.89±0.14]		104±7<br>[n=1.9±0.2]		204±16<br>[n=1.7±0.2]	
	1 mM Mg^{2+}	82±6<br>[n=1.47±0.12]		173±14<br>[n=1.4±0.2]		306±14<br>[n=1.36±0.08]	
k_{obs} (s^{-1}) [e]		1.46 ± 0.01		0.35 ± 0.01		0.59 ± 0.01	
k_{off} (s^{-1}) [f]	k_1 (contrib., %)	0.54 ± 0.01		0.74 ± 0.01 (68 ± 1)		1.20 ± 0.01 (83 ± 1)	
	k_2 (contrib., %)			0.07 ± 0.01 (32 ± 1)		0.12 ± 0.01 (17 ± 1)	
$t_{1/2}$ (s) [g]		1.3 ± 0.1		1.6 ± 0.1		0.75 ± 0.04	

[a] EGFP (QY=0.60 ref. [1]) and mTagBFP2 (QY=0.64 ref. [2]) were used as reference standards for 505-509- and 400-404-nm absorbing states, respectively. [b] Extinction coefficient was determined by alkaline denaturation. [c] Brightness was normalized to EGFP having QY of 0.60 and extinction coefficient of 55.3±3.6 $mM^{-1}cm^{-1}$. [d] Hill coefficient is shown in square brackets. [e] The observed association rates were determined at 300 nM Ca^{2+} concentration from association kinetics curves (ESI Figure S2). GCaMP6f and GCaMP6s had k_{obs} value of 1.28 ± 0.03 and 0.49 ± 0.05 sec^{-1}, respectively. [f] k_{off} values were estimated from calcium dissociation curves (ESI Figure S2) using mono or double exponential decay fitting with individual exponent contributions shown in the brackets. GCaMP6f and GCaMG6s had k_{off} values of 1.89 ± 0.01 and 0.69±0.01 s^{-1}, respectively. [g] GCaMP6f and GCaMP6s had t_{off} values of 0.37±0.04 and 1.01±0.04 s, respectively.

Table S2. Calcium association kinetics: k_{obs} rate constants at 700 and 1000 nM calcium concentration.

Indicator	k_{obs} (700 nM Ca^{2+}), s^{-1}		k_{obs} (1000 nM Ca^{2+}), s^{-1}	
	k_1 (contrib., %)	k_2 (contrib., %)	k_1 (contrib., %)	k_2 (contrib., %)
**NCaMP4**	5.15 ± 0.02		19.7 ± 0.3	
**NCaMP7**	2.1 ± 0.2 (88 ± 3%)	0.3 ± 0.1 (12 ± 3%)	7.0 ± 0.1 (72 ± 2%)	1.4 ± 0.1 (28 ± 2%)
**NCaMP9**	1.86±0.03 (83 ± 4%)	0.38±0.08 (17 ± 4%)	13.4 ± 0.9 (70 ± 10%)	4.7 ± 0.6 (30 ± 10%)
**NCaMP10**	1.35 ± 0.01		2.3 ± 0.1 (85 ± 10%)	1.4 ± 0.5 (15 ± 10%)
**GCaMP6s**	2.28 ± 0.01		5.99 ± 0.02	
**GCaMP6f**	6.36 ± 0.02		17.9 ± 0.2	

The observed association rates were determined at given Ca^{2+} concentrations from association kinetics curves (ESI Figure S2).

Table S3. Data collection, processing and refinement.

**Data collection**	
Diffraction source	K4.4 beamline, NRC "Kurchatov Institute"
Wavelength (Å)	0.79272
Temperature (K)	100
Detector	CCD
Crystal-to-detector distance (mm)	120.00
Rotation range per image (°)	1.0
Total rotation range (°)	276
Space group	$P2_12_12_1$
a, *b*, *c* (Å)	61.90; 65.70; 93.02
α, β, γ (°)	90.0
Average mosaicity (°)	0.124
Unique reflections	38947 (8372)
Resolution range (Å)	50.0-1.75 (1.90-1.75)
Completeness (%)	100.0 (100.0)
Average redundancy	11.4 (11.5)
$\langle I/\sigma(I)\rangle$	14.5 (3.4)
Rmeas (%)	16.5 (88.1)
$CC_{1/2}$	99.8 (85.0)
**Refinement**	
R_{fact} *(%)*	16.3
$R_{free.}$ *(%)*	19.3
Bonds (Å)	0.01
Angles (°)	1.81
Ramachandran plot	
Most favoured (%)	98.4
Allowed (%)	1.6
No. atoms	
Protein	3066
Water	357
Chromophore	19
Calcium ion	4
Other ligands	20
B-factors ($Å^2$)	
Protein	13.06
Water	25.31
Chromophore	14.55
Calcium ion	11.40
Other ligands	22.70

Values in parenthesis are for the highest-resolution shell.

Table S4. Comparison of the NCaMP7 and its variants response to the addition of ionomycin in HeLa cells.

Indicator in fusion	$\Delta F/F_0$	Relative $\Delta F/F_0$ [a]	Relative brightness[a]	N cells
**NES-jRGECO1a-P2A-NCaMP7**	10.0±2.4 (p=0.8016) [b]	3.2±0.9 (p=0.0079) [c]	1.1±0.2 (p=0.6667) [b]	5
**NES-jRGECO1a-P2A-NCaMP4**	2.0±0.6 (p=0.0079) [b]	1.0±0.5 (p=0.6032) [c]	1.3±0.4 (p=0.4127) [b]	
**NES-jRGECO1a-P2A-NCaMP9**	22.5±3.3 (p=0.0079) [b]	7.3±0.8 (p=0.0079) [c]	1.0±0.2 (p=0.8889) [b]	
**NES-jRGECO1a-P2A-NCaMP10**	19.6±1.7 (p=0.0159) [b]	7.2±0.9 (p=0.0079) [c]	1.4±0.2 (p=0.0238) [b]	
**NES-jRGECO1a-P2A-GCaMP6s**	12.2±3.1	4.4±1.2 (p=0.0079) [c]	1.0±0.3	

HeLa cells transiently co-expressing green NCaMPs and red NES-jRGECO1a indicators were imaged in DMEM medium supplemented with 20 mM HEPES, pH 7.40, 10% FBS, Glutamine, 50 U/ml penicillin, and 50 U/ml streptomycin before and after addition of 2.5 μM ionomycin till indicator fluorescence reached plateau. Errors correspond to standard deviations. Data were averaged across 5 cells.

[a] For estimation of relative $\Delta F/F_0$ and brightness values, the maximal $\Delta F/F_0$ values and maximal green fluorescence of calcium indicators were normalized to the maximal $\Delta F/F_0$ values and maximal red fluorescence of jRGECO1a red calcium indicator co-expressing in the same cell. Relative brightness was additionally normalized to the relative brightness of GCaMP6s.

[b] p value shows statistical difference between the respective values for NCaMPs and GCaMP6s control indicator.

[c] p value shows statistical difference between the respective values for NCaMPs/GCaMP6s and jRGECO1a control indicator.

Table S5. Characteristics of calcium responses to external field stimulation of neurons co-expressing the green NCaMPs and GCaMP6s indicators with control red RGECO1 GECI in dissociated neuronal culture.

Indicator	$\Delta F/F_0$/1AP, % [b]	N cells
**NCaMP7**	40±9 ($p<0.0001$)	8
**NCaMP4**	19±13 ($p=0.2494$)	5
**NCaMP9**	36±9 ($p<0.0001$)	8
**NCaMP10**	13±18 ($p=0.0953$)	9
**GCaMP6s** [a]	13±8	44

Neurons expressing NES-R-GECO1-P2A-NCaMPs were stimulated and imaged on DIV 7th using confocal microscope and electric stimulator as described earlier [3]. Neuronal cultures were transfected on DIV 4th using calcium-phosphate approach. Errors correspond to standard deviations. Data were averaged across 5-44 cells.

[a] Neurons co-expressing the GCaMP6s and NES-R-GECO1 indicators were imaged and stimulated on DIV 14-20th. Neuronal cultures were transduced on DIV 4th with the mixture of rAAVs viral particles carrying GCaMP6s and NES-R-GECO1.

[b] The $\Delta F/F$ values normalized to 1AP were calculated as a slope of the dependence of $\Delta F/F$ responses vs a number of APs (ESI Figure S7). A number of APs was calculated according to the $\Delta F/F$ response of red R-GECO1 indicator co-expressing in the same cell, assuming that its $\Delta F/F$ value of 4±1% corresponds to 1AP and has linear dependence from a number of APs [4]. p value shows statistical difference between the respective values for NCaMPs and GCaMP6s control indicator.

Table S6. Comparison of calcium responses in neurons expressing NCaMPs indicators in hippocampus of freely moving mice registered with nVista HD miniscope *in vivo*.

Properties	Indicators			
	NCaMP4	NCaMP7	NCaMP9	NCaMP10
**Number of mice**	2	1	2	1
**Number of active cells** [a]	220+180	51	236+176	112
**Number of spikes detected**	1491	202	1049	1050
**Rise half-time, s** [b]	0.9±0.6	0.9±0.7	1.2±0.8	0.8±0.6
**Decay half-time, s** [b]	2.3±1.3	2.1±1.2	2.5±1.2	2.0±1.2
**Peak ΔF/F_0** [c]	0.012±0.008	0.017±0.015	0.013±0.009	0.016±0.014
**SNR** [d]	4.8±2.7	8.7±6.6	4.6±2.7	5.1±2.0

[a] Cell was considered as active, if at least one spike exceeding 4MAD threshold was registered.
[b] Rise and decay half-times were calculated as time intervals between the peak of the mean spike and half-peak at front and back slopes of the spike.
[c] Peak ΔF/F_0 was calculated as $(F-F_0)/F_0$, where F_0 is the baseline fluorescence signal averaged over the whole period of imaging.
[d] Signal-to-noise ratio (SNR) was quantified as peak ΔF/F_0 response over median absolute deviation (MAD), calculated for each cell over the whole period of imaging.
[b-d] Mean values ± standard error of mean are given. Analysis of calcium imaging data was performed as previously described [3].

Table S7. Comparison of calcium responses in neurons expressing NCaMP7 and GCaMP6s indicators in hippocampus of freely moving mice registered with nVista HD miniscope *in vivo*.

Properties	Indicators	
	NCaMP7	GCaMP6s
**A number of mice**	2	4
**A number of active cells** [a]	230	436
**A number of spikes detected**	325	316
**Rise half-time, s** [b]	0.95±0.52 ($p<0.0001$)	0.76±0.49
**Decay half-time, s** [b]	3.0±0.9 ($p<0.0001$)	2.5±0.9
**Peak $\Delta F/F_0$** [c]	1.9±1.6 ($p=0.0615$)	2.1±1.8

[a] Only spikes exceeding 4MAD threshold and not less than 50% of maximum value of the trace were taken into account.
[b] Rise and decay half-times were calculated as time intervals between the peak of the mean spike and half-peak at front and back slopes of the spike.
[c] Peak $\Delta F/F_0$ is given in MIN1PIPE units, which are $\Delta F/F_0$ units after normalization and registration.
[b-c] Mean values ± standard error of mean are given. Analysis of calcium imaging data was performed as previously described [3].
p value shows statistical difference between the respective values for NCaMP7 and GCaMP6s control indicator.

Table S8. List of primers.

Primer	Primer sequence (5′-3′)
**Neon-BglII-2**	CGAGATCTATGGTCTCAAAGGGAGAGGAG
**Neon-EcoRI-r2**	GCGAATTCCTACTTATACAGCTCGTCCATGC
**NEON-M13:**	GACAAATTCCCTGACAGCCNNSNNSNNSTCATCACGTCGTAAGTGG.
**Neon-M13-r:**	CCACTTACGACGTGATGASNNSNNSNNGGCTGTCAGGGAATTTGTC
**M13-CaM**	CTATAGGTCGGCTGAGCTCAGGCGGTAGCGACCAACTGACTGAAGAG
**M13-CaM-r**	CTCTTCAGTCAGTTGGTCGCTACCGCCTGAGCTCAGCCGACCTATAG
**CaM-Neon**	CAAATGATGACAGCGAAGNNSNNSNNSGCTGACTGGTGTAGATCC
**CaM-Neon-r**	GGATCTACACCAGTCAGCSNNSNNSNNCTTCGCTGTCATCATTTG
**Neon-CaM**	GACAAATTCCCTGACAGCCNNSNNSNNSGACCAACTGACTGAAGAGC
**Neon-CaM-r**	GCTCTTCAGTCAGTTGGTCSNNSNNSNNGGCTGTCAGGGAATTTGTC
**CaM-M13**	CAAATGATGACAGCGAAGGGCGGTAGCTCATCACGTCGTAAGTGG
**CaM-M13-r**	CCACTTACGACGTGATGAGCTACCGCCCTTCGCTGTCATCATTTG
**M13-Neon**	CTATAGGTCGGCTGAGCTCANNSNNSNNSGCTGACTGGTGTAGATCC
**M13-Neon-r**	GGATCTACACCAGTCAGCSNNSNNSNNTGAGCTCAGCCGACCTATAG
**GCaMP-BglII**	CGAGATCTGCTAGCATGGTCGACTCATCACGTC
**GCaMP-EcoRI-HindIII-r**	GTCAAGCTTGAATTCCTACTTCGCTGTCATCATTTG

```
        1       10        20        30        40        50        60
        |        |         |         |         |         |         |
Library MVSKGEEDNMASLPATHELHIFGSINGVDFDMVGQGTGNPNDGYEELNLKSTKGDLQFSP
NCaMP7  MVSKGEEENMASLPATHELHIFGSINGIDFDMVGQGTGNPNDGYEELNLKSTMGDLQFSP
NCaMP4  MVSKGEEENMASLPATHELHIFGSINGIDFDMVGQGTGNPNDGYEELNLKSTMGDLQFSP
NCaMP9  MVSKGEEENMASLPATHELHIFGSINGIDFDMVGQGTGNPNDGYEELNLKSTMGDLQFSP
NCaMP10 MVSKGEEENMASLPATHELHIFGSINGIDFDMVGQGTGNPNDGYEELNLKSTMGDLQFSP

       61       70        80        90       100       110       120
        |        |         |         |         |         |         |
Library WILVPHIGYGFHQYLPYPDGMSPFQAAMVDGSGYQVHRTMQFEDGASLTVNYRYTYEGSH
NCaMP7  WILVPHIGYGFHQYLPYPDGMSPFQAAMVDGSGYQVHRTMQFEDGASLTVNYRYTYEGSH
NCaMP4  WILVPHIGYGFHQYLPYPDGMSPFQAAMVDGSGYQVHRTMQFEDGASLTVNYRYTYEGSH
NCaMP9  WILVPHIGYGFHQYLPYPDGMSPFQAAMVDGSGYQVHRTMQFEDGASLTVNYRYTYEGSH
NCaMP10 WILVPHIGYGFHQYLPYPDGMSPFQAAMVDGSGYQVHRTMQFEDGASLTVNYRYTYEGSH

      121      130       140       150       160       170       180
        |        |         |         |         |         |         |
Library IKGEAQVKGTGFPADGPVMTNSLTAXXXDQLTEEQIAEFKEAFSLFDKDGDGTITTKELG
NCaMP7  IKGEAQVEGTGFPADGPVMTNSLTAEAHDQLTEEQIAEFKEAFSLFDKDGDGTITTKELG
NCaMP4  IKGEAQVEGTGFPADGPVMTNSLTAEAHDQLTEEQIAEFKEAFSLFDKDGDGTITTKELG
NCaMP9  IKGEAQVEGTGFPADGPVMTNSLTAEAHDQLTEEQLAEFKEAFSLFDKDGDGTITTKELG
NCaMP10 IKGEAQVEGTGFPADGPVMANSLTAEAHDQLTEEQIAEFKEAFSLFDKDGDGTITTKELG
                                                 * * * *     *

      181      190       200       210       220       230       240
        |        |         |         |         |         |         |
Library TVMRSLGQNPTEAELQDMINEVDADGDGTIDFPEFLTMMARKMKYRDTEEEIREAFGVFD
NCaMP7  TVMRSLGQNPTEAELRVMIIEVDADGDGTLDFPEFLAMMARKMKYRDTEEEIREAFGVFD
NCaMP4  TVMRSLGQSPTEAELRVMINEVDADGDGTLDFPEFLTMMARKMKYRDTEEEIREAFGVFD
NCaMP9  TVMRSLGQNPTEAELRVMINEVDADGDGTLDFPEFLTMMARKMKYRDTEEEIREAFGVFD
NCaMP10 TVMRSLGQNPTEAELRVMINEVDADGDGTLDFPGFLTMMARKMKYRDTEEEIREAFGVFD
                              * * * * *  *                      *

      241      250       260       270       280       290       300
        |        |         |         |         |         |         |
Library KDGNGYISAAELRHVMTNLGEKLTDEEVDEMIREADIDGDGQVNYEEFVQMMTAKGGSSS
NCaMP7  KDGNGYIGAAELRHVMTNLGEKLTDEEVGELIREADIDGDGQVNYEEFVQMMTAKGGSGG
NCaMP4  KDGNGYIGAAELRHVMTNLGEKLTDEEVGELIREADIDGDGQVNYEEFVQMMTAKGGSGG
NCaMP9  KDGNGYIGAAELRHVMTNLGEKLTDEEVGELIREADIDGDGQVNYEEFVQMMTAKGGSGG
NCaMP10 KDGNGYIGAAELRHVLTNLGEKLTDEEVGELIREADIDGDGQVNYEEFVQMMTAKGGSGG
         * * *    *                          * * * *     *

      301      310       320       330       340       350       360
        |        |         |         |         |         |         |
Library GSSSRRKWNKTGHAVRAIGRLSSXXXADWCRSKKTYPNDKTIISTFKWSYTTGNGKRYRS
NCaMP7  GSSSRRKWNKAGHAVRAIGRLSSMYFADWCVSKKTCPNDKTIVSTFKWAFITDNGKRYRS
NCaMP4  GSSSRRKWNKAGHAVRAIGRLSSMYFADWCVSKKTYPNDKTIVSTLKWAFITDNGKRYRS
NCaMP9  GSSSRRKWNKAGHAVRAIGRLSSMYFADWCVSKKTYPNDKTIVSAFKWAFITDNGKRYRS
NCaMP10 GSSSRRKWNKAGHAVRAIGRLSSMYFADWCVSKKTYPNDKTIVSTFKWAFITDNGKRYRS

      361      370       380       390       400       410
        |        |         |         |         |         |         |
Library TARTTYTFAKPMAANYLKNQPMYVFRKTELKHSKTELNFKEWQKAFTDVMGDELYK
NCaMP7  TARTTYTFAKPMAANYLKNQPMYVFRKTELKHSKTELNFKEWQKAFTDVMGDELYK
NCaMP4  TARTTYTFAKPMAANYLKNQPMYVFRKTELKHSKTELNFKEWQKAFTDVMGDELYK
NCaMP9  TARTTYTFAKPMAANYLKNQPMYVFRKTELKHSKTELNFKEWQKAFTDVMGDELYK
NCaMP10 TARTTYTFAKPMAANYLKNQPMYVFRKTELKHSKTELNFKEWQKAFTDVMGDELYK
```

Figure S1. Alignment of the amino acid sequences for the original library and NCaMP calcium indicators. Alignment numbering follows that of NCaMPs. Residues from fluorescent part buried in β-can are highlighted with green. Residues that are forming chromophore are underlined. Mutations in NCaMPs related to the original library including linkers between fluorescent and calcium-binding parts are highlighted in red. Residues that are forming Ca^{2+}-binding loops are highlighted in cyan. Calcium-coordinating residues are selected with asterisks.

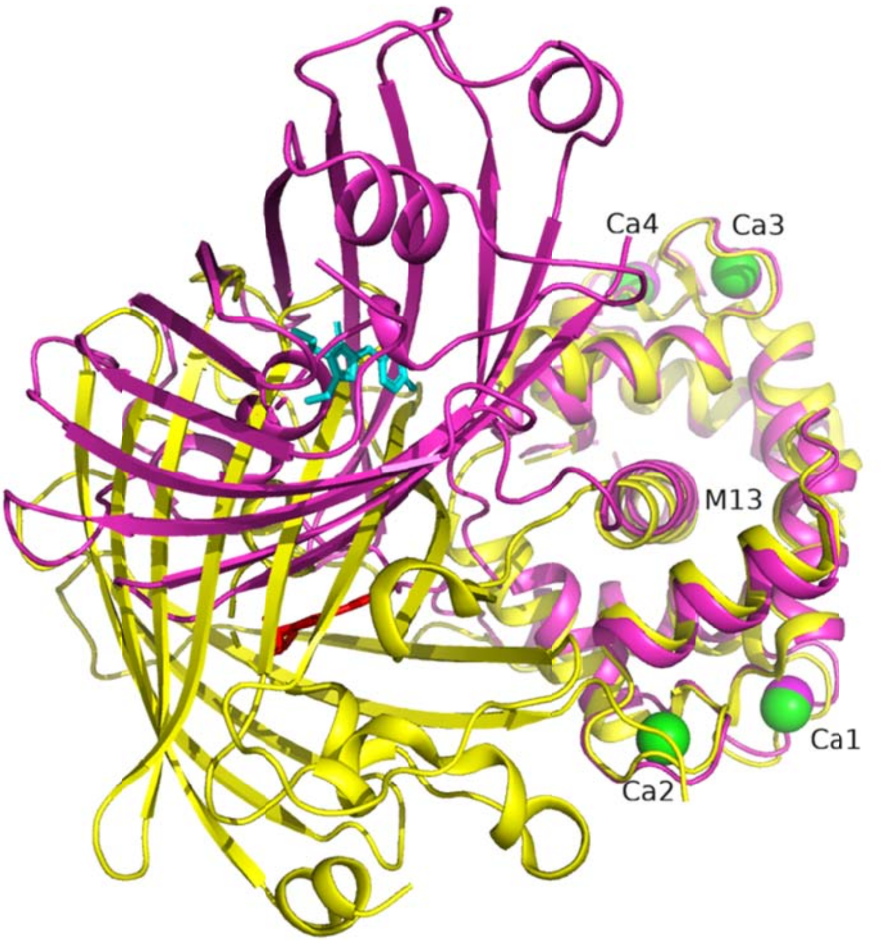

Figure S2. Superposition of NCaMP7 (PDB ID - 6XW2, in magenta) and GCaMP6m (PDB ID 3WLD, in yellow) structures. Chromophores for NCaMP7 and GCaMP6m are shown in cyan and red colors, respectively. Calcium ions for NCaMP7 and GCaMP6m are in magenta and green. M13 peptide is labeled as well as calcium ions. Structures are superposed by their calmodulin domains.

```
            160       170       180       190       200       210
GCaMP6m       MVSKGEELFTGVVPILVELDGDVNGHKFSVSGEGEGDATYGKLTLKFICTTGKLPVPW
NCaMP7      MVSKGEEENMASLPATHELHIFGSINGIDFDMVGQGTGNPNDGYEELNLKSTMGDLQFSP
            1       10        20        30        40        50        60

            220       230       240       250       260       270
GCaMP6m     PTLVTTLTYGVQCFSRYPDHMKQHDFFKSAMPEGYIQERTIFFKDDGNYKTRAEVKFEGD
NCaMP7      WILVPHIGYGFHQYLPYPDGMS-PFQAAMVDGSGYQVHRTMQFEDGASLTVNYRYTYEGS
                        70        80         90       100       110

            280       290       300       310       320       330
GCaMP6m     TLVNRIELKGIDFKEDGNILGHKLEYN-LPDQLTEEQIAEFKEAFSLFDKDGDGTITTKE
NCaMP7      HIKGEAQVEGTGFPADGPVMTNSLT-AEAHDQLTEEQIAEFKEAFSLFDKDGDGTITTKE
                                                             * * * *     *
            120       130       140         150       160       170

            340       350       360       370       380       390
GCaMP6m     LGTVMRSLGQNPTEAELQDMINEVDADGDGTIDFPEFLTMMARKGSYRDTEEEIREAFGV
NCaMP7      LGTVMRSLGQNPTEAELRVMIIEVDADGDGTLDFPEFLAMMARKMKYRDTEEEIREAFGV
                                    * * * * *   *
             180       190       200       210       220       230

            400       410       420       430       440       450
GCaMP6m     FDKDGNGYISAAELRHVMTNLGEKLTDEEVDEMIREADIDGDGQVNYEEFVQMMTAKGGT
NCaMP7      FDKDGNGYIGAAELRHVMTNLGEKLTDEEVGELIREADIDGDGQVNYEEFVQMMTAKGGS
             * * * *     *                          * * * *     *
             240       250       260       270       280       290

             40        50        60             70        80
GCaMP6m     GG---SRRKWNKTGHAVRAIGRLSSLE-----NVYIKADKQKNGIKANFKIRHNIEDGGV
NCaMP7      GGGSSSRRKWNKAGHAVRAIGRLSSMYFADWCVSKKTCP-NDKTIVSTFKWAFITDNGKR
             300       310       320       330        340       350

            90        100         110       120       130       140
GCaMP6m     QLAYHYQQNTPIGDG---PVLLPDNHYLSVQSKLSKDPNEKRDHMVLLEFVTAAGITLGM
NCaMP7      YRSTARTTYTFAKPMAANYLKNQP-MYVFRKTELKHS----KTELNFKEWQKAFTDVMGM
             360       370       380         390          400       410

              150
GCaMP6m     DELYK
NCaMP7      DELYK
```

Figure S3. Alignment of the amino acid sequences for the GCaMP6m and NCaMP7 fluorescent proteins. Alignment numbering follows that of GCaMP6m (top line) and NCaMP7 (bottom line). Residues from fluorescent part buried in β-can are highlighted with green. Residues that are forming chromophore are underlined. Mutations in NCaMP7 appeared during protein engineering including linkers between fluorescent and calcium-binding parts are highlighted in red. Residues that are forming Ca^{2+}-binding loops are highlighted in cyan. Calcium-coordinating residues are selected with asterisks. Secondary structure is presented for the NCaMP7 indicator based on its X-ray data.

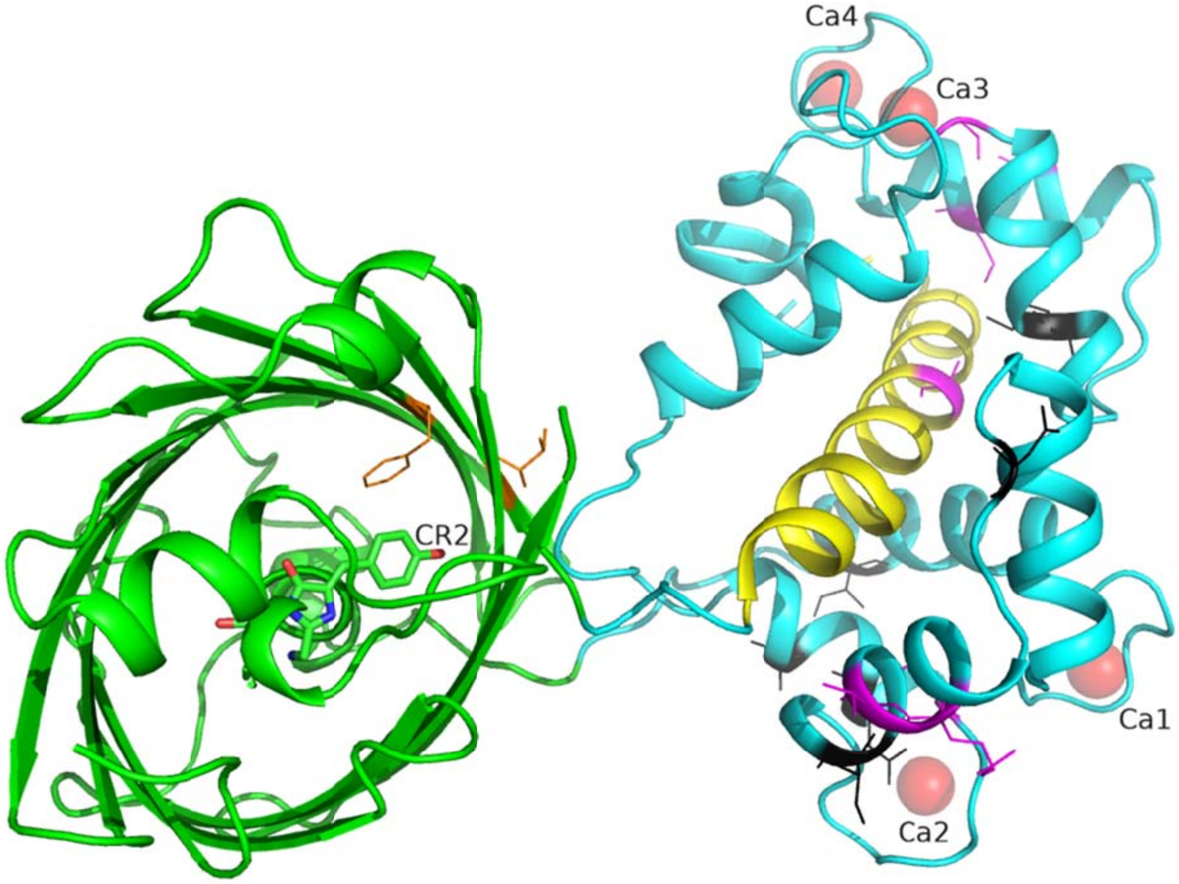

Figure S4. Overview of the mutations found during mutagenesis of NCaMPs on the crystal structure of the NCaMP7 indicator (PDB ID - 6XW2). Cartoon representation of NCaMP7 crystal structure with mutations in fluorescent domain close to the chromophore (within 6Å, in orange), common mutation in CaM and M13-peptide calcium-binding domain (in magenta) and mutations unique to each of NCaMPs (in black).

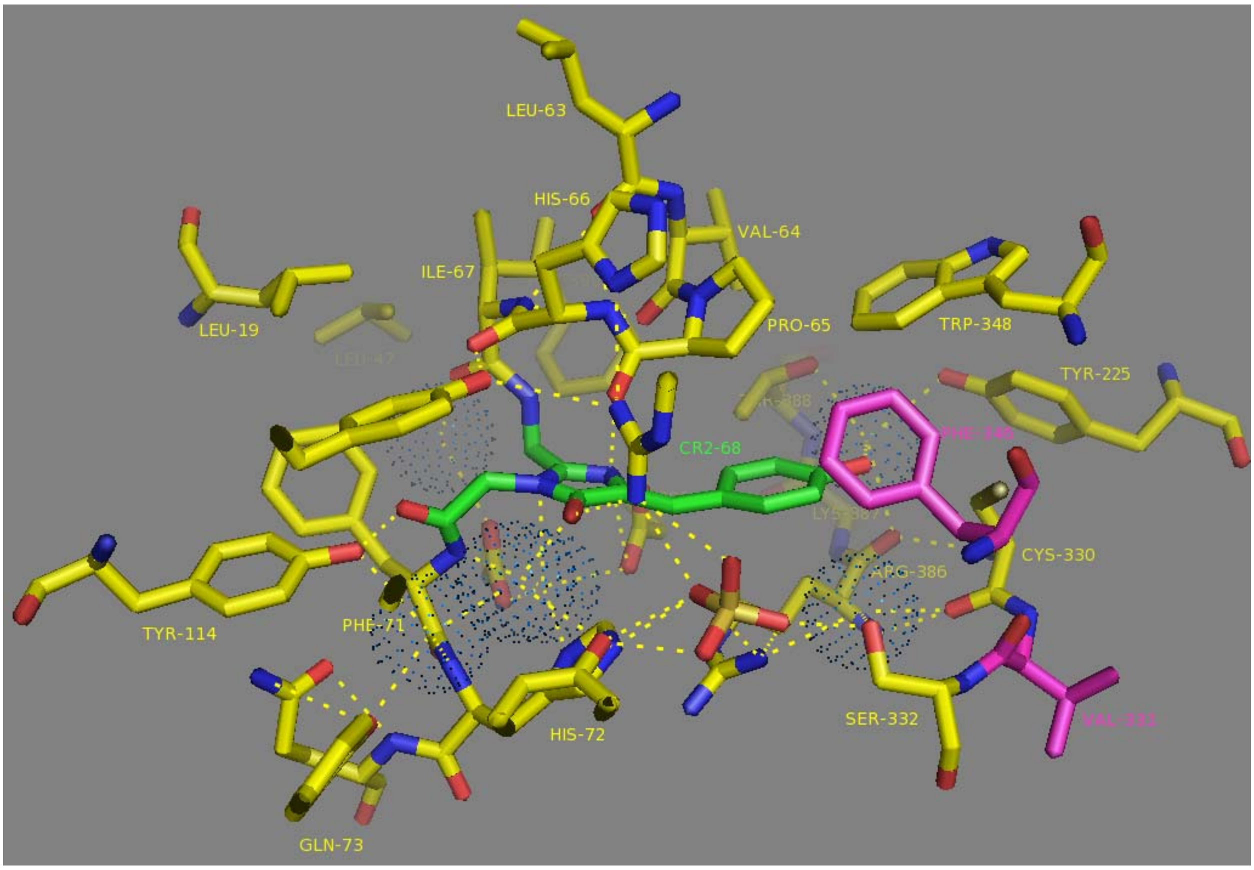

Figure S5. 6.0Å chromophore environment according to NCaMP7 X-ray structure (PDB ID - 6XW2). Chromophore, 6.0Å-surrounding residues, water molecules as dots and V331, F346 residues are shown in green, yellow, blue and magenta colors, respectively. H-bonds are shown as dash lines.

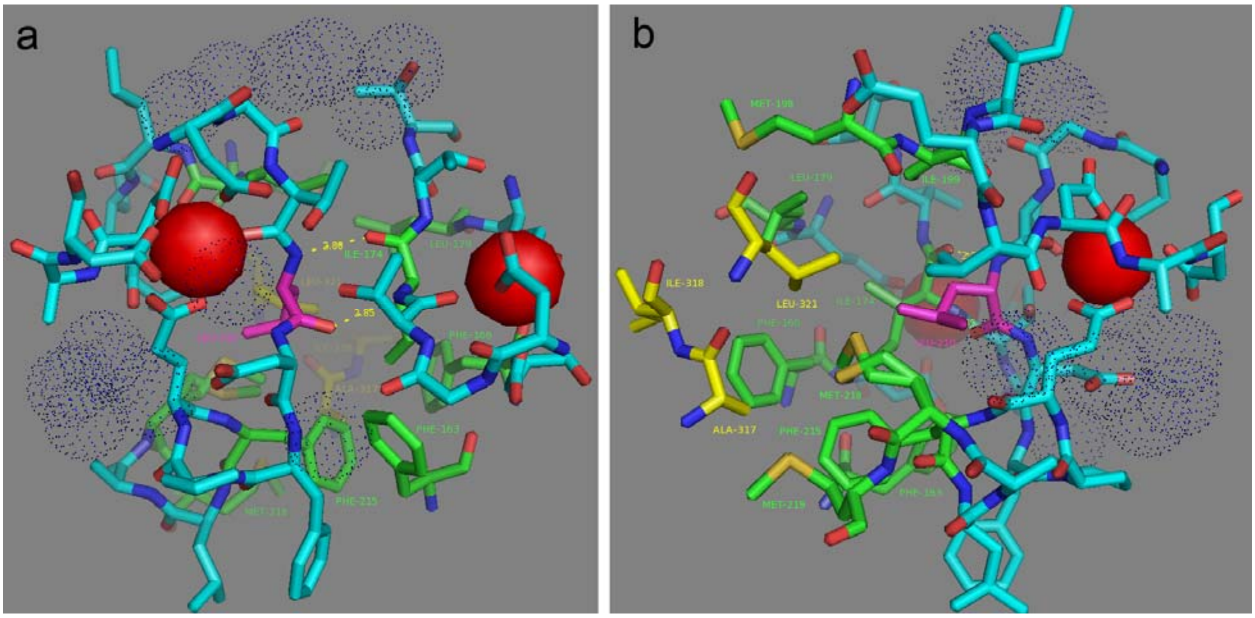

Figure S6. L210 residue hydrogen (a) and hydrophobic (b) contacts according to NCaMP7 X-ray structure (PDB ID - 6XW2). (a) Two H-bonds between L210 (in magenta) and I174 residues are shown as dash lines. **(b)** Side group of L210 residue (in magenta) is exposed to the hydrophobic pocket which is formed by residues from M13-peptide (A317, I318, and L321, in yellow) and CaM (EF1 and EF2 hands and adjacent to them amino acids) (F163, F166, I174, L179, I199, L210, F215, M218, and M219, in green). Calcium ions and water molecules are shown as red spheres and blue dots, respectively.

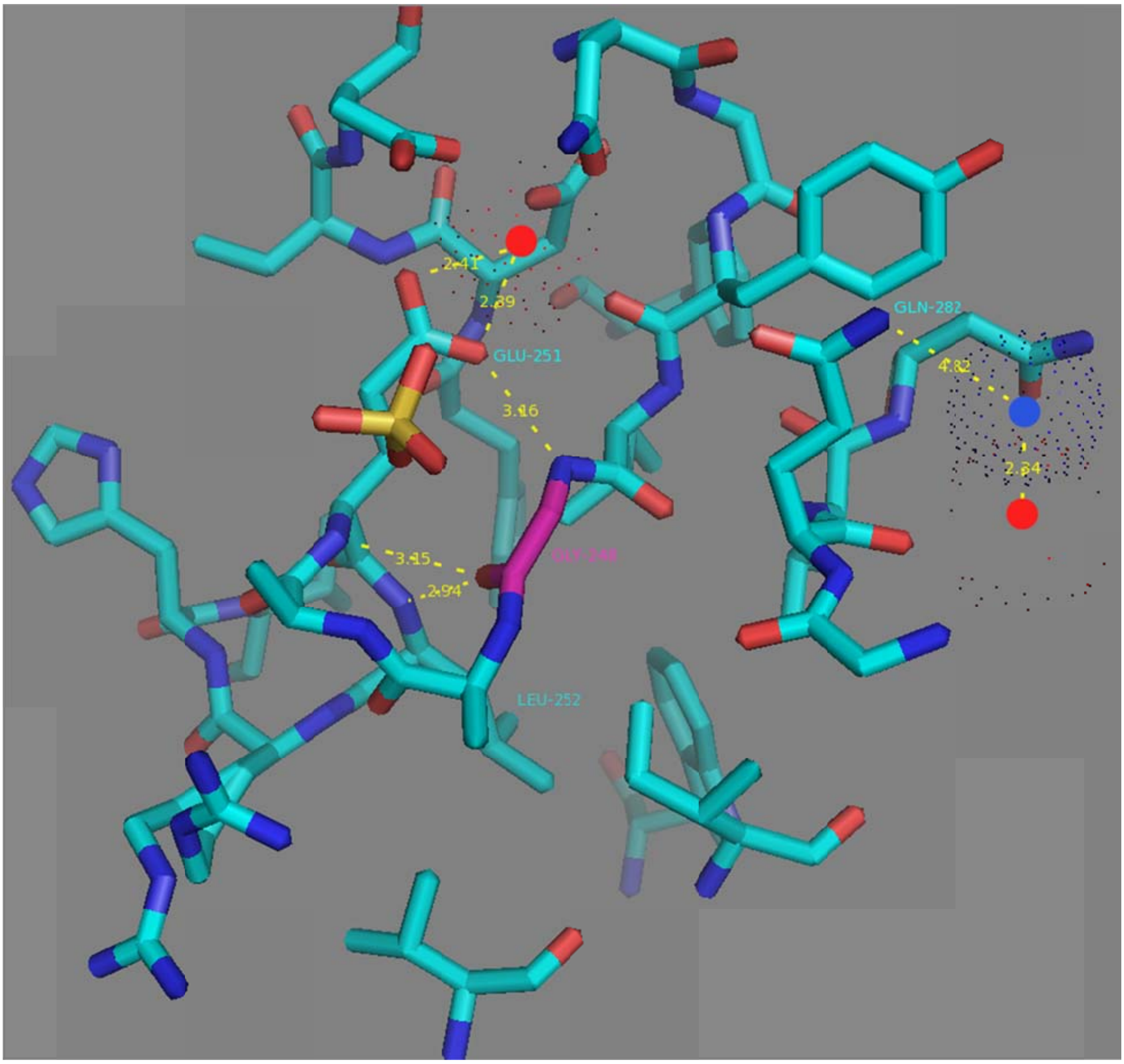

Figure S7. G248 residue surrounding according to NCaMP7 X-ray structure (PDB ID - 6XW2). H-bonds are shown as dash lines between G248 (in magenta) and E251, E251 and calcium ion from EF3 (as red dots), G248 and L252, Q282 and water molecule (as blue dots), water molecule and calcium ion from EF4 (as red dots).

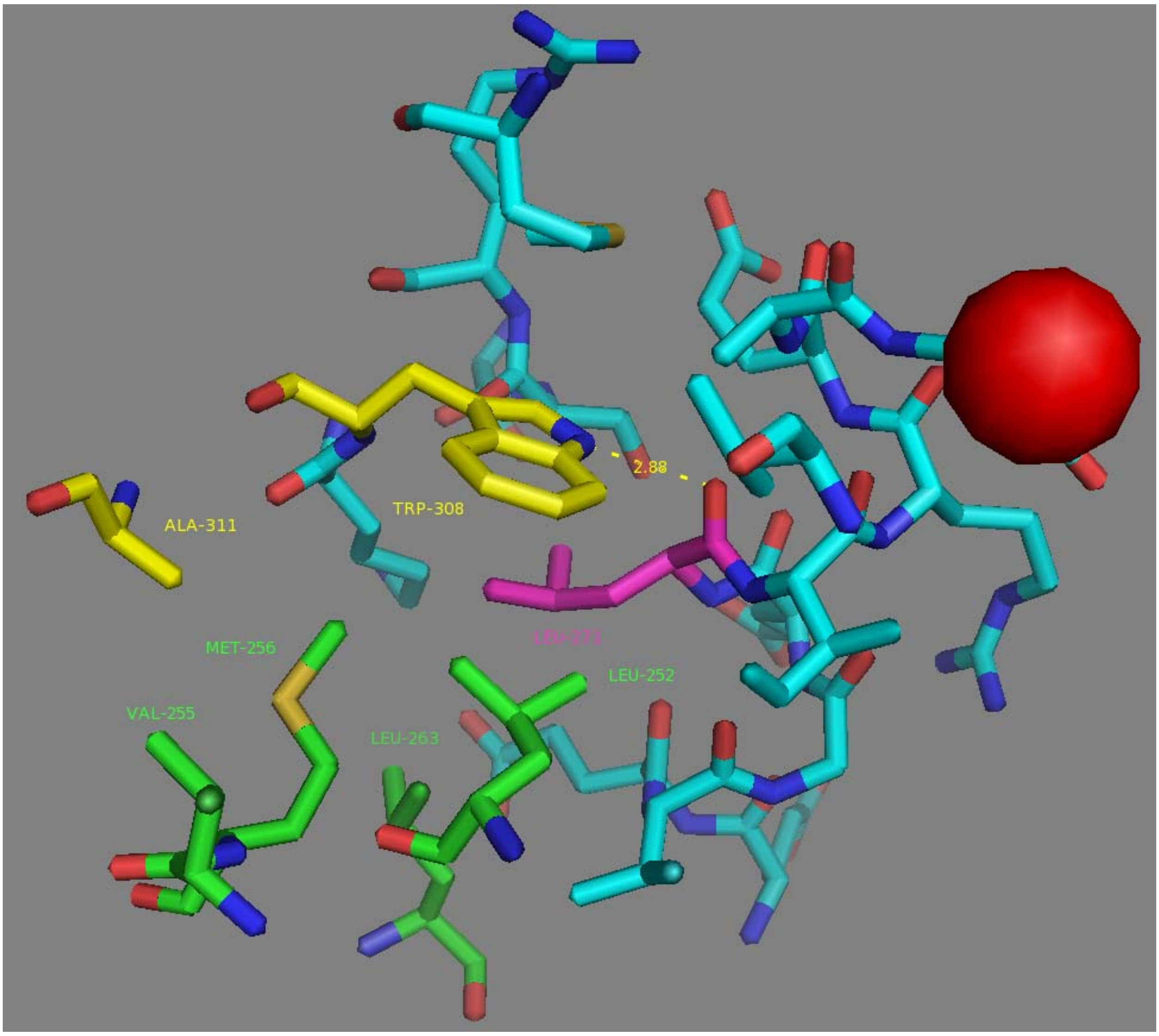

Figure S8. L271 residue surrounding according to NCaMP7 X-ray structure (PDB ID - 6XW2). H-bond is shown as dash lines between L271 (in magenta) and W308. E251 Calcium ion from EF4 is shown as red sphere. Side group of L271 residue (in magenta) is exposed to the hydrophobic pocket which is formed by residues from M13-peptide (A311 and W30, in yellow) and CaM (L252, V255, M256, L263 and L271, in green).

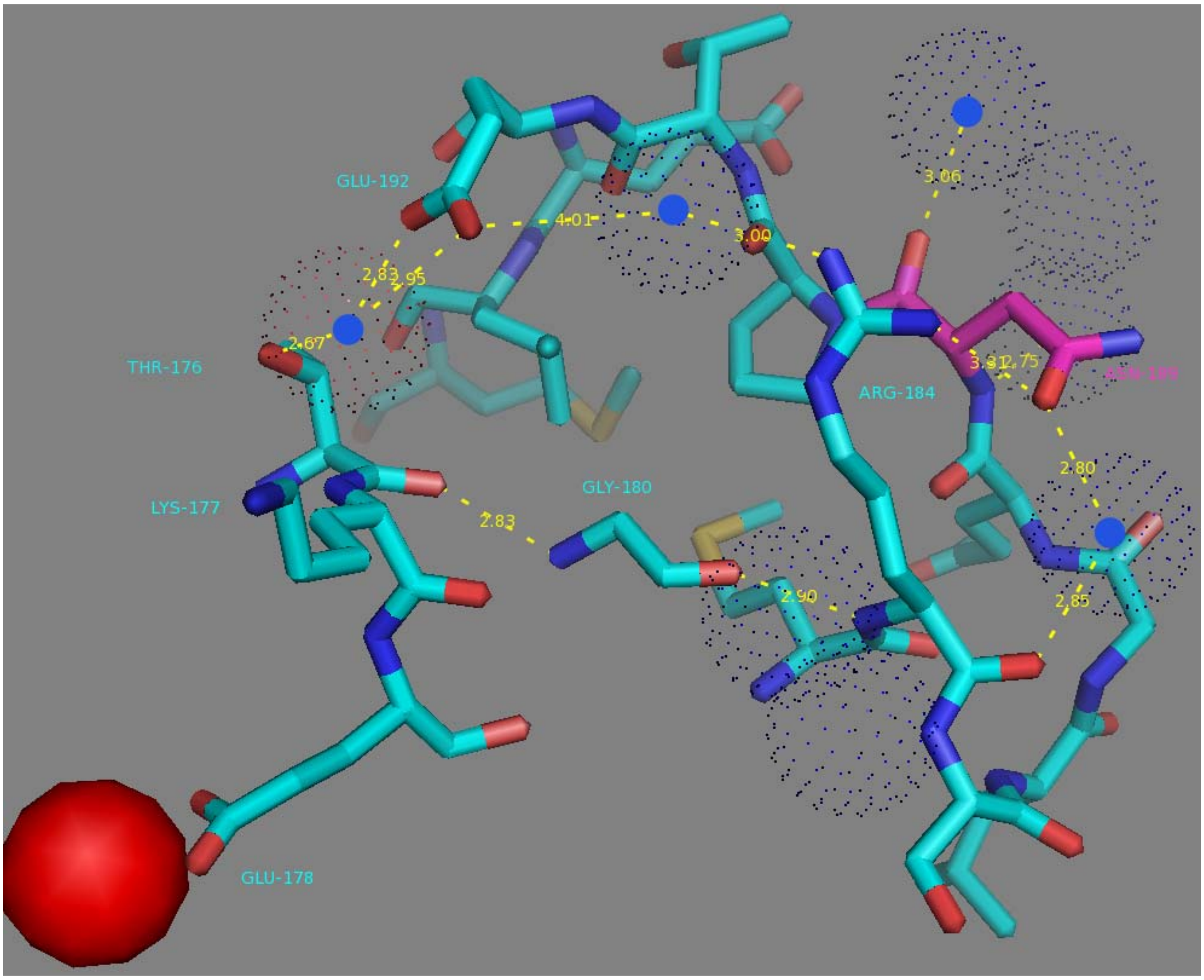

Figure S9. N189 residue surrounding and H-bonds network to the T176 residue from EF1 calcium coordinating center according to NCaMP7 X-ray structure (PDB ID - 6XW2). H-bonds network is shown as dash lines between N189 (in magenta), R184, water molecules (in blue dots), E192, G180 and T176. Calcium ion from EF1 is shown as red sphere.

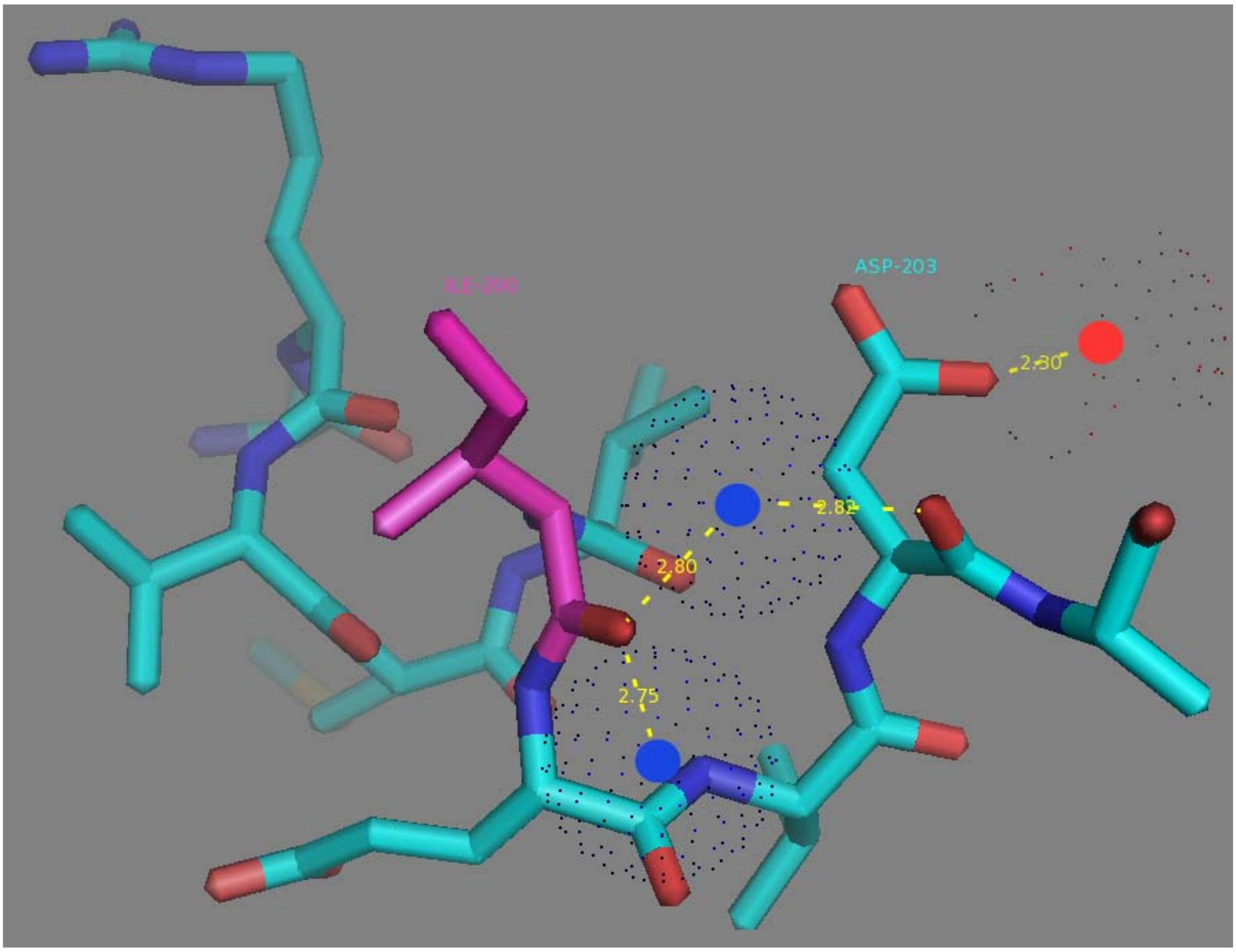

Figure S10. I200 residue surrounding and H-bonds network to the D203 residue from EF2 calcium coordinating center according to NCaMP7 X-ray structure (PDB ID - 6XW2). H-bonds network is shown as dash lines between I200 (in magenta), water molecules (in blue dots), E203 and calcium ion from EF2 (red sphere).

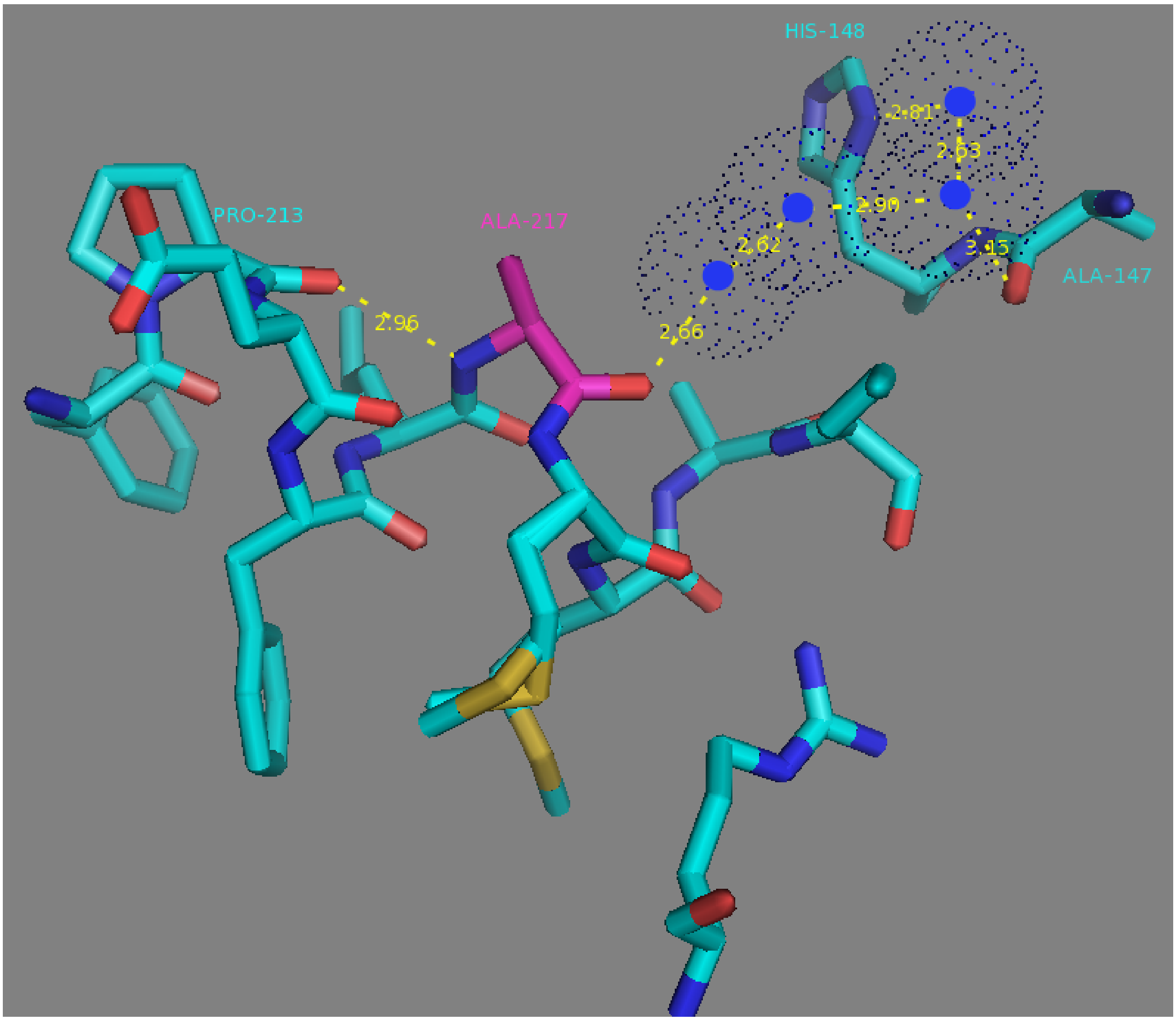

Figure S11. A217 residue surrounding and H-bonds network to the A147 and H148 residues from linker between fluorescent and calcium-binding domains according to NCaMP7 X-ray structure (PDB ID - 6XW2). H-bonds network is shown as dash lines between A217 (in magenta), water molecules (in blue dots), P213, A147 and H148.

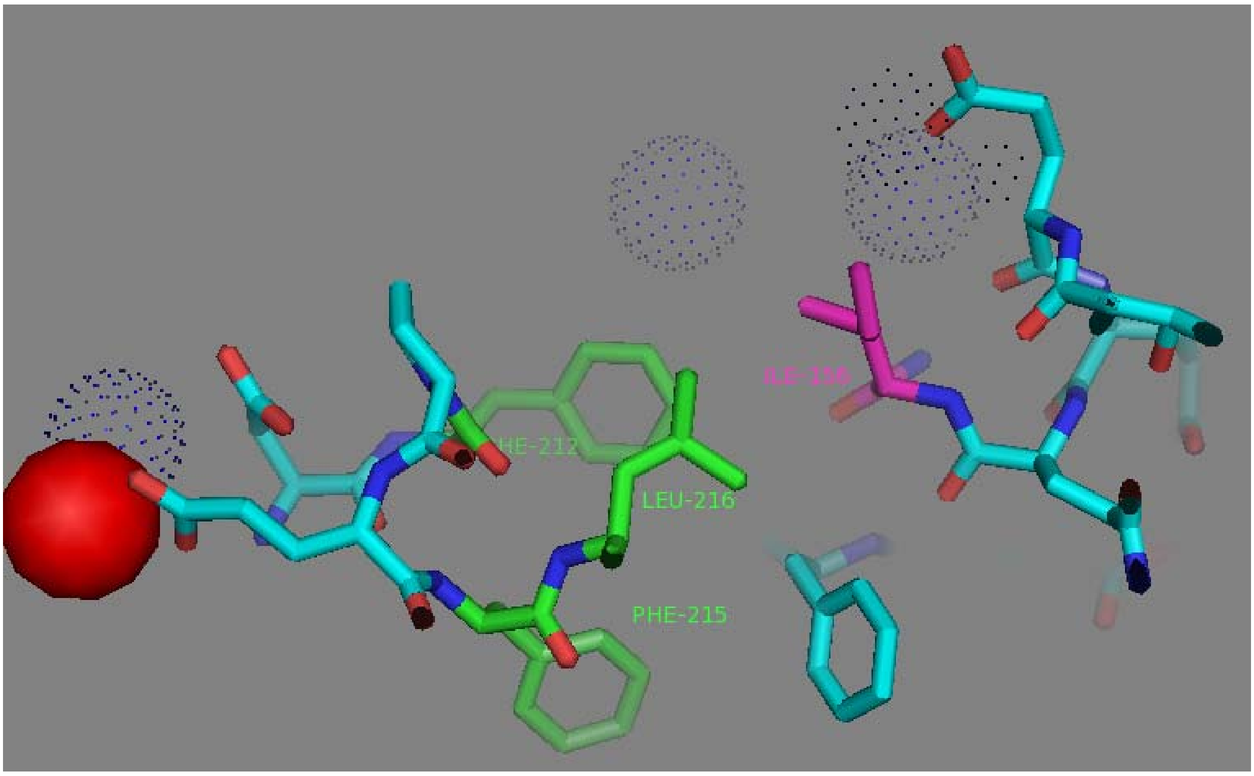

Figure S12. I156 residue surrounding and network to the residues in EF2 coordinating calcium ion according to NCaMP7 X-ray structure (PDB ID - 6XW2). Calcium ion from EF2 is shown as red sphere. Side group of I156 residue (in magenta) is exposed to the hydrophobic pocket which is formed by residues from EF2 (F212, in green) and adjacent to EF2 amino acids (F215 and L216, in green). Water molecules are shown in blue dots.

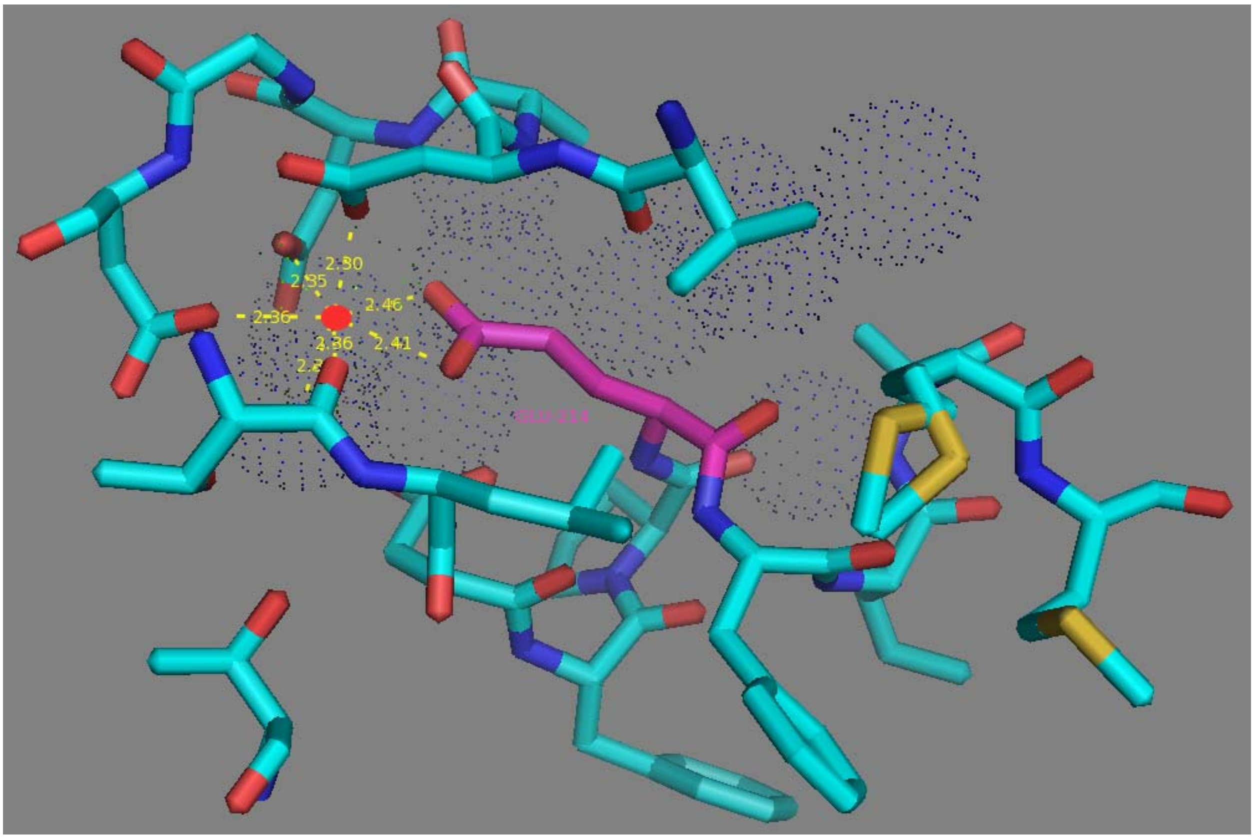

Figure S13. E214 residue surrounding and calcium coordination sphere in EF2 hand according to NCaMP7 X-ray structure (PDB ID - 6XW2). Side group of E214 residue (in magenta) forms two H-bonds with calcium ion (as red dots) from EF2. Water molecules are shown as blue dots.

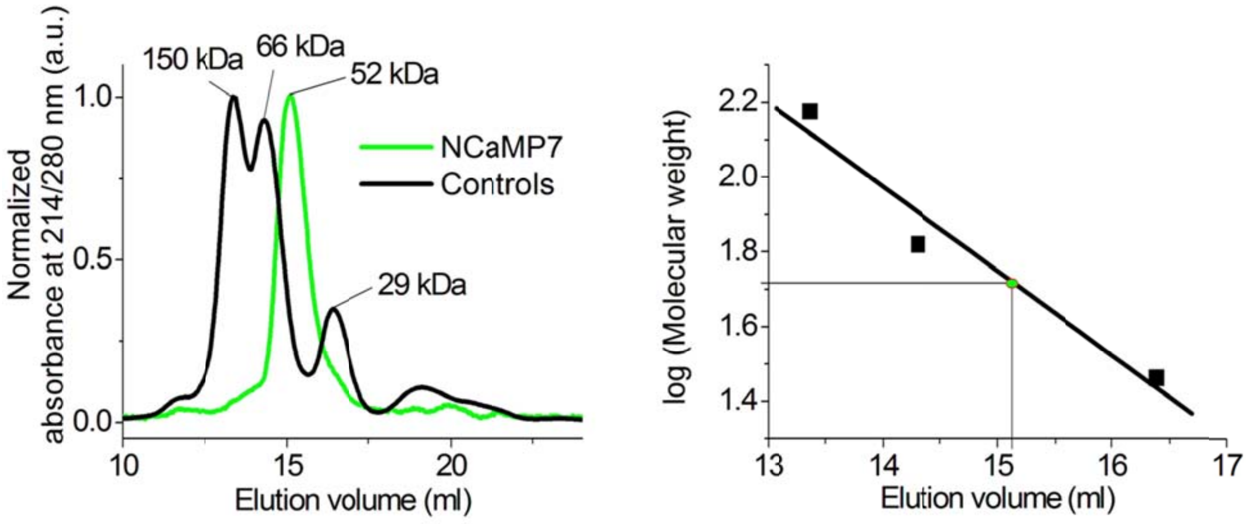

Figure S14. Fast protein liquid chromatography of NCaMP7. NCaMP7 (1.7 mg/ml) was eluted in 40 mM Tris-HCl (pH 7.5) and 200 mM NaCl buffer supplemented with 5 mM $CaCl_2$. The molecular weight of NCaMP7 was determined from a linear regression of the dependence of logarithm of control molecular weights vs elution volume.

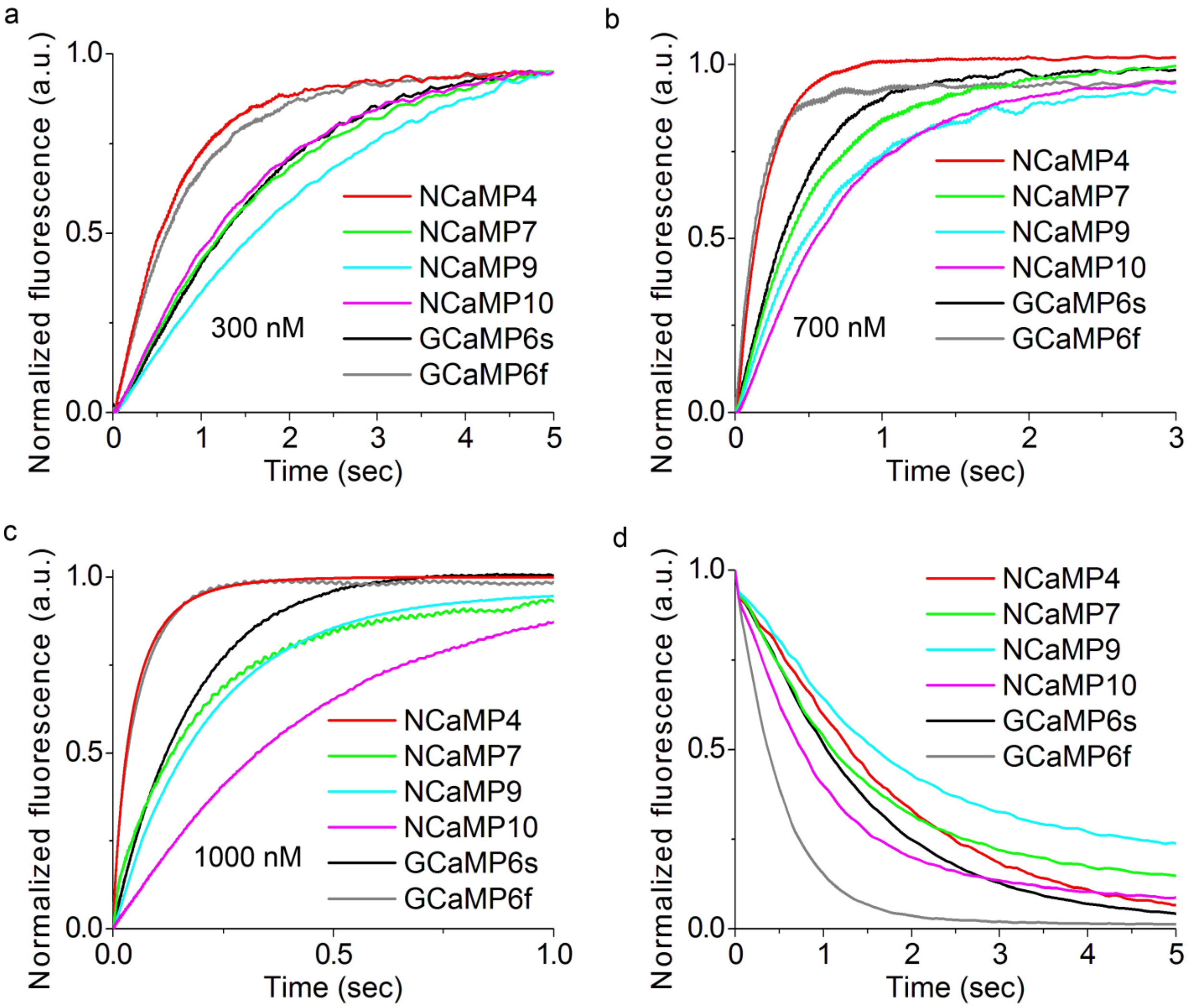

Figure S15. Calcium-association and -dissociation kinetics for the NCaMP7 indicator, its variants, GCaMP6s and GCaMP6f investigated using stopped-flow fluorimetry. (a)-(c) Calcium-association kinetics curves for given GECIs were recorded at 300, 700 and 1000 nM Ca^{2+}-free final concentration. **(d)** Calcium-dissociation kinetics for respective GECIs was recorded at 1000 nM Ca^{2+}-free starting concentration. Three replicates were averaged for analysis.

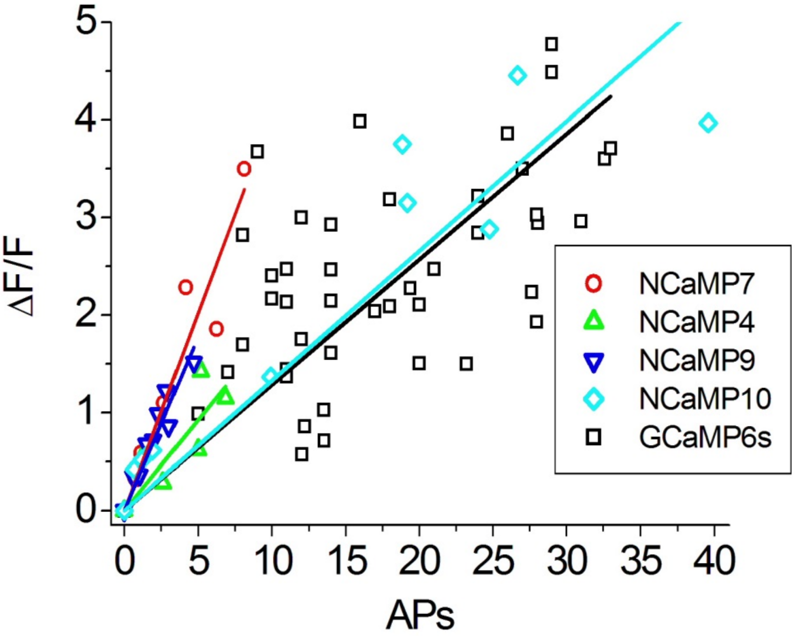

Figure S16. Comparison of responses of green NCaMPs and GCaMP6s indicators normalized to the response of the control red R-GECO1 GECI to external field stimulation of neurons co-expressing the GECIs in dissociated neuronal culture. The numeric values of the slopes calculated from the dependences of ΔF/F over a number of APs are listed in ESI Table S5.

NES-NCaMP7-stop gene:
ATGCTTCAACTTCCTCCTCTTGAACGTCTTACTCTTTCGAGATCTATGGTCTCAAAGGGAGAGGAG
GAAAACATGGCTAGTCTGCCTGCTACCCACGAACTGCATATTTTTGGCTCCATAAATGGCATCGA
TTTCGACATGGTAGGCCAAGGCACCGGTAATCCAAACGACGGCTATGAGGAGCTGAACCTGAAG
TCCACCATGGGCGATCTGCAGTTCAGTCCCTGGATCCTGGTGCCGCACATTGGGTACGGGTTTCA
CCAGTATCTTCCCTATCCAGACGGGATGTCACCATTTCAGGCTGCAATGGTCGACGGGTCCGGAT
ACCAAGTGCACCGCACTATGCAGTTTGAGGACGGTGCCTCACTGACCGTGAATTACAGATACAC
CTACGAAGGATCACATATCAAAGGGGAAGCTCAGGTGGAGGGCACTGGGTTCCCAGCTGACGG
CCCTGTGATGACAAATTCCCTGACAGCCGAGGCCCACGACCAGCTGACTGAAGAGCAGATCGCT
GAATTTAAAGAGGCTTTCTCCCTATTTGACAAGGACGGGGATGGGACAATAACAACCAAGGAGC
TGGGGACGGTGATGCGGTCTCTGGGGCAGAACCCCACAGAAGCAGAGCTGCGGGTCATGATCAT
TGAAGTAGATGCCGACGGTGACGGCACACTCGACTTCCCTGAGTTCCTGGCAATGATGGCAAGA
AAAATGAAATACAGGGACACGGAAGAAGAAATTAGAGAAGCGTTCGGTGTGTTTGATAAGGAT
GGCAATGGCTACATCGGTGCAGCAGAGCTTCGCCATGTGATGACAAACCTTGGAGAGAAGTTAA
CAGATGAGGAGGTTGGTGAATTGATCAGGGAAGCAGACATCGATGGGGATGGTCAGGTGAACT
ACGAGGAGTTTGTACAAATGATGACAGCGAAGGGCGGTTCAGGAGGCGGGAGCTCATCACGTC
GTAAGTGGAATAAGGCAGGTCACGCAGTCAGAGCCATAGGTCGGCTGAGCTCAATGTACTTCGC
TGACTGGTGCGTATCCAAGAAGACTTGCCCCAATGATAAGACTATCGTCTCCACATTTAAGTGGG
CTTTTATCACCGACAACGGCAAACGCTACCGGTCTACCGCTCGCACCACATACACATTCGCCAAG
CCAATGGCCGCAAATTACCTCAAAAATCAGCCGATGTATGTCTTTCGGAAAACAGAGCTGAAAC
ATTCTAAGACCGAACTGAATTTTAAAGAGTGGCAAAAGGCCTTTACGGACGTGATGGGCATGGA
CGAGCTGTATAAGTAA

NES-NCaMP4-stop gene:
ATGCTTCAACTTCCTCCTCTTGAACGTCTTACTCTTTCGAGATCTATGGTCTCAAAGGGAGAGGAG
GAAAACATGGCTAGTCTGCCTGCTACCCACGAACTGCATATTTTTGGCTCCATAAATGGCATCGA
TTTCGACATGGTAGGCCAAGGCACCGGTAATCCAAACGACGGCTATGAGGAGCTGAACCTGAAG
TCCACCATGGGCGATCTGCAGTTCAGTCCCTGGATCCTGGTGCCGCACATTGGGTACGGGTTTCA
CCAGTATCTTCCCTATCCAGACGGGATGTCACCATTTCAGGCTGCAATGGTCGACGGGTCCGGAT
ATCAAGTGCACCGCACTATGCAGTTTGAGGACGGTGCTTCACTGACCGTGAATTACAGATACACC
TACGAAGGATCACATATCAAAGGGGAAGCTCAGGTGGAGGGCACTGGGTTCCCAGCTGACGGC
CCTGTGATGACAAATTCCCTGACAGCCGAGGCCCACGACCAGCTGACTGAAGAGCAGATCGCTG
AATTTAAAGAGGCTTTCTCCCTATTTGACAAGGACGGGGATGGGACAATAACAACCAAGGAGCT
GGGGACGGTGATGCGGTCTCTGGGGCAGAGCCCCACAGAAGCAGAGCTGCGGGTCATGATCAAT
GAAGTAGATGCCGACGGTGACGGCACACTCGACTTCCCTGAGTTCCTGACAATGATGGCAAGAA
AAATGAAATACAGGGACACGGAAGAAGAAATTAGAGAAGCGTTCGGTGTGTTTGATAAGGATG
GCAATGGCTACATCGGTGCAGCAGAGCTTCGCCATGTGATGACAAACCTTGGAGAGAAGTTAAC
AGATGAAGAGGTTGGTGAATTGATCAGGGAAGCAGACATCGATGGGGATGGTCAGGTGAACTA
CGAGGAGTTTGTACAAATGATGACAGCGAAGGGCGGTTCAGGAGGCGGGAGCTCATCACGTCGT
AAGTGGAATAAGGCAGGTCACGCAGTCAGAGCCATAGGTCGGCTGAGCTCAATGTACTTCGCTG
ACTGGTGCGTATCCAAGAAGACTTACCCCAATGATAAGACTATCGTCTCCACACTTAAGTGGGCT
TTTATCACCGACAACGGCAAACGCTACCGGTCTACCGCTCGCACCACATACACATTCGCCAAGC
CAATGGCCGCAAATTACCTCAAAAATCAGCCGATGTATGTCTTTCGGAAAACTGAGCTGAAACA
TTCTAAGACCGAACTGAATTTTAAAGAGTGGCAAAAGGCCTTTACGGACGTGATGGGCATGGAC
GAGCTGTATAAGTAA

NES-NCaMP9-stop gene:
ATGCTTCAACTTCCTCCTCTTGAACGTCTTACTCTTTCGAGATCTATGGTCTCAAAGGGAGAGGAG
GAAAACATGGCTAGTCTGCCTGCTACCCACGAACTGCATATTTTTGGCTCCATAAATGGCATCGA
TTTCGACATGGTAGGCCAAGGCACCGGTAATCCAAACGACGGCTATGAGGAGCTGAACCTGAAG
TCCACCATGGGCGATCTGCAGTTCAGTCCCTGGATCCTGGTGCCGCACATTGGGTACGGGTTTCA
CCAGTATCTTCCCTATCCAGACGGGATGTCACCATTTCAGGCTGCAATGGTCGACGGGTCCGGAT
ATCAAGTGCACCGCACTATGCAGTTTGAGGACGGTGCTTCACTGACCGTGAATTACAGATACACC
TACGAAGGATCACATATCAAAGGGGAAGCTCAGGTGGAGGGCACTGGGTTCCCAGCTGACGGC
CCTGTGATGACAAATTCCCTGACAGCCGAGGCCCACGACCAGCTGACTGAAGAGCAGCTCGCTG
AATTTAAAGAGGCTTTCTCCCTATTTGACAAGGACGGGGATGGGACAATAACAACCAAGGAGCT
GGGGACGGTGATGCGGTCTCTGGGGCAGAACCCCACAGAAGCAGAGCTGCGGGTCATGATCAAT

GAAGTAGATGCCGACGGTGACGGCACGCTCGACTTCCCTGAGTTCCTGACAATGATGGCAAGAA
AAATGAAATACAGGGACACGGAAGAAGAAATTAGAGAAGCGTTCGGTGTGTTTGATAAGGATG
GCAATGGCTACATCGGTGCAGCAGAGCTTCGCCATGTGATGACAAACCTTGGAGAGAAGTTAAC
AGATGAAGAGGTTGGTGAATTGATCAGGGAAGCAGACATCGATGGGGATGGTCAGGTGAACTA
CGAGGAGTTTGTACAAATGATGACAGCGAAGGGCGGTTCAGGAGGCGGGAGCTCATCACGTCGT
AAGTGGAATAAGGCAGGTCACGCAGTCAGAGCCATAGGTCGGCTGAGCTCAATGTACTTCGCTG
ACTGGTGCGTATCCAAGAAGACTTACCCCAATGATAAGACTATCGTCTCCGCATTTAAGTGGGCT
TTTATCACCGACAACGGCAAACGCTACCGGTCTACCGCTCGCACCACATACACATTCGCCAAGC
CAATGGCCGCAAATTACCTCAAAAATCAGCCGATGTATGTCTTTCGGAAAACAGAGCTGAAACA
TTCTAAGACCGAACTGAATTTTAAAGAGTGGCAAAAGGCCTTTACGGACGTGATGGGCATGGAC
GAGCTGTATAAGTAA

NES-NCaMP10-stop gene:

ATGCTTCAACTTCCTCCTCTTGAACGTCTTACTCTTTCGAGATCTATGGTCTCAAAGGGAGAGGAG
GAAAACATGGCTAGTCTGCCTGCTACCCACGAACTGCATATTTTTGGCTCCATAAATGGCATCGA
TTTCGACATGGTAGGCCAAGGCACCGGTAATCCAAACGACGGCTATGAGGAGCTGAACCTGAAG
TCCACCATGGGCGATCTGCAGTTCAGTCCCTGGATCCTGGTGCCGCACATTGGGTACGGGTTTCA
CCAGTATCTTCCCTATCCAGACGGGATGTCACCATTTCAGGCTGCAATGGTCGACGGGTCCGGAT
ATCAAGTGCACCGCACTATGCAGTTTGAGGACGGTGCTTCACTGACCGTGAATTACAGATACACC
TACGAAGGATCACATATCAAAGGGGAAGCTCAGGTGGAGGGCACTGGGTTCCCAGCTGACGGC
CCTGTGATGGCAAATTCCCTGACAGCCGAGGCCCACGACCAGCTGACTGAAGAGCAGATCGCTG
AATTTAAAGAGGCTTTCTCCCTATTTGACAAGGACGGGGATGGGACAATAACAACCAAGGAGCT
GGGGACGGTGATGCGGTCTCTGGGGCAGAACCCCACAGAAGCAGAGCTGCGGGTCATGATCAAT
GAAGTAGATGCCGACGGTGACGGCACACTCGACTTCCCTGGGTTCCTGACAATGATGGCAAGAA
AAATGAAATACAGGGACACGGAAGAAGAAATTAGAGAAGCGTTCGGTGTGTTTGATAAGGATG
GCAATGGCTACATCGGTGCAGCAGAGCTTCGCCATGTGTTGACAAACCTTGGAGAGAAGTTAAC
AGATGAAGAGGTTGGTGAATTGATCAGGGAAGCAGACATCGATGGGGATGGTCAGGTGAACTA
CGAGGAGTTCGTACAAATGATGACAGCGAAGGGCGGTTCAGGAGGCGGGAGCTCATCACGTCGT
AAGTGGAATAAGGCAGGTCACGCAGTCAGAGCCATAGGTCGGCTGAGCTCAATGTACTTCGCTG
ACTGGTGCGTATCCAAGAAGACTTACCCCAATGATAAGACTATCGTCTCCACATTTAAGTGGGCT
TTTATCACCGACAACGGCAAACGCTACCGGTCTACCGCTCGCACCACATACACATTCGCCAAGC
CAATGGCCGCAAATTACCTCAAAAATCAGCCGATGTATGTCTTTCGGAAAACAGAGCTGAAACA
TTCTAAGACCGAACTGAATTTTAAAGAGTGGCAAAAGGCCTTTACGGACGTGATGGGCATGGAC
GAGCTGTATAAGTAA

Figure S17. Nucleotide sequences for NES-NCaMPs indicators. NES – nuclei exclusion signal.

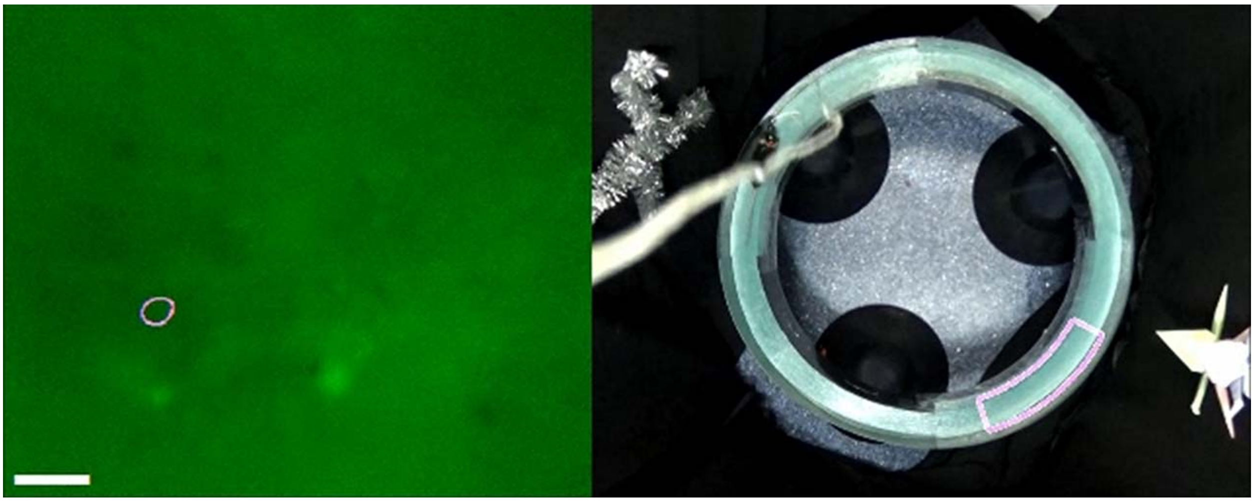

Video S1. ***In vivo*** **specific neuronal place cell activity in the CA1 area of hippocampus of mouse visualized with the NCaMP7 calcium indicator and nVista HD miniscope synchronized with mouse position**. A video of specific neuronal place cell calcium activity in CA1 area of mouse hippocampus displayed as ΔF/F changes in fluorescence at 20 Hz frame rate (left) synchronized with mouse movement in O-shaped track (right). GLP 1040 lens probe was used. Video is shown for a time from 4.5 to 6.5 min. Scalebar, 100 μm. The identified place cell is marked by circle and corresponds to the cell shown in Main text, Figure 5e.

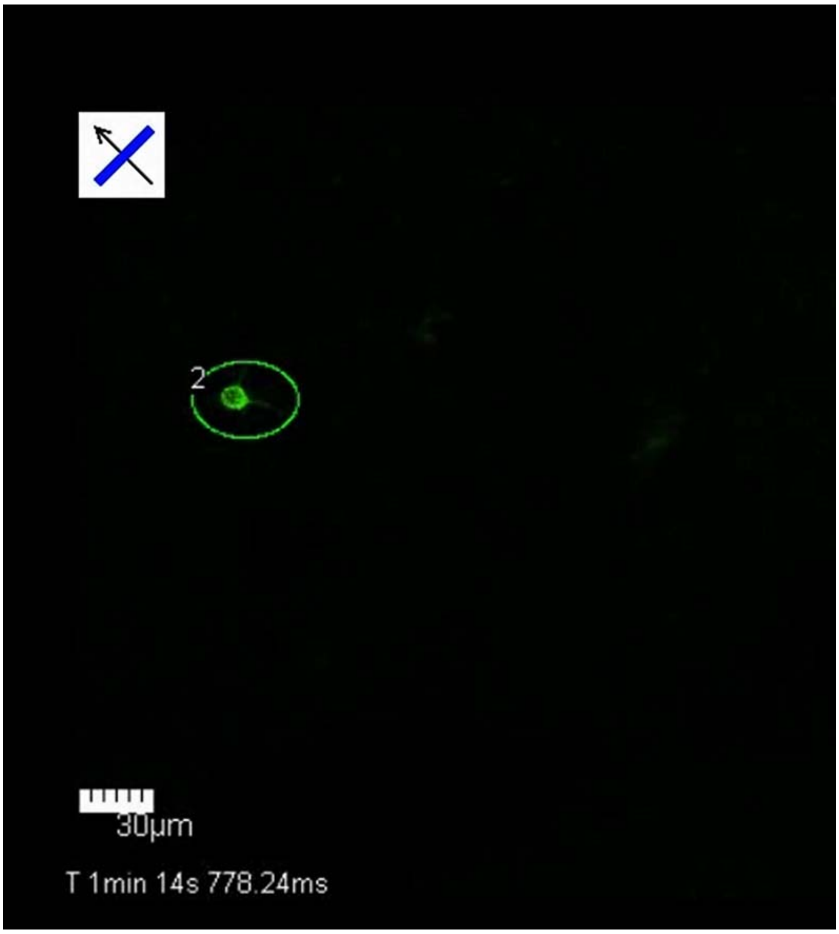

Video S2. ***In vivo*** **moving grating-evoked neuronal calcium activity in the mouse V1 visual cortex at a 104 µm depth as visualized with the NCaMP7 calcium indicator and two-photon microscopy.** Single frame for the time of 1m14s778.24ms is shown. Neurons specifically activated by the moving grating visual stimulus (shown as blue; the direction of grating is shown with black arrow) are selected with green circle. This field of view corresponds to the neuron 2 shown in the main text, Figure 5b.

Supplementary references

1. Tsien, R. Y., The green fluorescent protein. *Annu Rev Biochem* **1998,** 67, 509-44.
2. Subach, O. M.; Cranfill, P. J.; Davidson, M. W.; Verkhusha, V. V., An enhanced monomeric blue fluorescent protein with the high chemical stability of the chromophore. *PloS one* **2011,** 6, (12), e28674.
3. Barykina, N. V.; Doronin, D. A.; Subach, O. M.; Sotskov, V. P.; Plusnin, V. V.; Ivleva, O. A.; Gruzdeva, A. M.; Kunitsyna, T. A.; Ivashkina, O. I.; Lazutkin, A. A.; Malyshev, A. Y.; Smirnov, I. V.; Varizhuk, A. M.; Pozmogova, G. E.; Piatkevich, K. D.; Anokhin, K. V.; Enikolopov, G.; Subach, F. V., NTnC-like genetically encoded calcium indicator with a positive and enhanced response and fast kinetics. *Sci Rep* **2018,** 8, (1), 15233.
4. Wu, J.; Abdelfattah, A. S.; Miraucourt, L. S.; Kutsarova, E.; Ruangkittisakul, A.; Zhou, H.; Ballanyi, K.; Wicks, G.; Drobizhev, M.; Rebane, A.; Ruthazer, E. S.; Campbell, R. E., A long Stokes shift red fluorescent Ca(2+) indicator protein for two-photon and ratiometric imaging. *Nat Commun* **2014,** 5, 5262.
